# A social-ecological analysis of community perceptions of dengue fever and *Aedes aegypti* in Machala, Ecuador

**DOI:** 10.1186/1471-2458-14-1135

**Published:** 2014-11-04

**Authors:** Anna M Stewart Ibarra, Valerie A Luzadis, Mercy J Borbor Cordova, Mercy Silva, Tania Ordoñez, Efraín Beltrán Ayala, Sadie J Ryan

**Affiliations:** Center for Global Health and Translational Science and Department of Microbiology & Immunology, State University of New York Upstate Medical University, Syracuse, NY USA; Department of Environmental Studies, State University of New York College of Environmental Science and Forestry, Syracuse, NY USA; Escuela Superior Politécnica del Litoral, Guayaquil, Guayas Ecuador; Ministerio de Salud Pública, Machala, El Oro Ecuador; Facultad de Medicina, Universidad Técnica de Machala, Machala, El Oro Ecuador; Department of Geography and Emerging Pathogens Institute, University of Florida, Gainesville, FL USA; School of Life Sciences, College of Agriculture, Engineering, and Science, University of KwaZulu-Natal, Durban, South Africa

**Keywords:** Dengue fever, *Aedes aegypti*, Community perceptions, Vector control, Ecuador, Social-ecological systems

## Abstract

**Background:**

The growing burden of dengue fever and the lack of a vaccine or specific medical treatment have increased the urgency of the public health sector to identify alternative management strategies. A prevailing trend in Latin America has been a shift towards decentralized vector control programs with integrated management strategies, requiring significant intersectoral coordination, community engagement, and knowledge of the local social-ecological system (SES). Community perceptions and responses are a critical component of this system, since perceptions shape actions, and thus govern behavioral responses and acceptance of shifts in policy and management.

**Methods:**

We investigated perceptions, misconceptions, and local SES risk factors for dengue in high risk communities located at the urban periphery and center in Machala, Ecuador. We facilitated twelve focus group discussions with community members using semi-structured question guides and causal diagrams. Focus groups were recorded, transcribed, and coded to identify emergent themes using qualitative methods for theme analysis. To estimate the relative importance of the themes in each study area, we tabulated the number of focus groups in which each theme was present. Household surveys (n = 79) were conducted to further explore these themes, and we compared survey responses from the two areas using descriptive statistics.

**Results:**

We identified thirty biophysical, political-institutional, and community-household risk factors for dengue. People at the periphery identified a greater number of risk factors. Dengue control required considerable investment of time and resources, which presented a greater challenge for women and people at the periphery. Common misperceptions included confusion with other febrile diseases, lack of knowledge of transmission mechanisms, and misconceptions about mosquito behavior. People perceived that dengue control programs had been limited by the lack of inter-institutional coordination and lack of social cohesion.

**Conclusions:**

There is a need for local, policy-relevant research that can be translated to strengthen the design, implementation, and evaluation of new dengue management strategies. This study contributes to a growing body of research in this area. Based on these findings, we identify key policy and management recommendations that will inform the ongoing transition to a decentralized dengue control program in Ecuador and other dengue endemic countries.

**Electronic supplementary material:**

The online version of this article (doi:10.1186/1471-2458-14-1135) contains supplementary material, which is available to authorized users.

## Background

Dengue fever, a mosquito-borne febrile viral illness, continues to increase in severity, incidence, and distribution in Latin America and the Caribbean [1]. Over 8.4 million cases were reported in the Americas from 2000 to 2010, a dramatic increase from the 2.7 million cases reported in the 1990s [2]. Until a vaccine becomes available, control of *Aedes aegypti* and *Aedes albopictus* mosquitoes remains the principal means of preventing and managing dengue outbreaks. However, the sustainability of traditional vector control strategies is threatened by the high demand for materials (larvicide, adult insecticides), trained field personnel, and the high frequency of household visits required in endemic regions.

**Figure 1:**
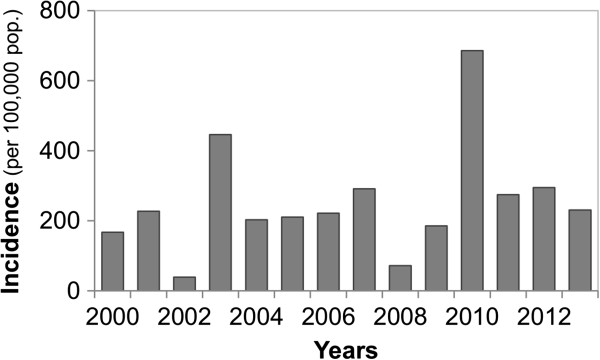
**Annual dengue fever incidence in El Oro Province, 2000–2013 [** [6] **].**

In Ecuador, dengue has replaced malaria as the most prevalent mosquito-borne disease [3]. More than 100,000 cases of dengue have been reported from Ecuador over the last decade, principally from the lowland coastal region, where the disease is hyper-endemic [3]. Notably, Machala, Ecuador, the site of this study, had the highest *Ae. aegypti* indices in recent multi-country studies in Asia and Latin America [4, 5]. In 2010, southern coastal Ecuador experienced the largest dengue epidemic on record (Figure 1). The epidemic began in the city of Machala, El Oro province, where 2,019 cases of dengue fever and 77 cases of dengue hemorrhagic fever (DHF) were reported, resulting in incidence rates of 83.6 cases of dengue and 3.2 cases of DHF per 10,000 population. People under the age of twenty bore the greatest burden of disease, accounting for 58% of all reported cases [6, 7].

The growing burden of dengue, the lack of a vaccine or specific medical treatment, and the high cost of vector control have increased the urgency of the public health sector to identify alternative management strategies. The Pan American Health Organization and others have supported a shift from traditional vertical control programs towards decentralized vector control programs with integrated management strategies in dengue endemic regions [8–11]. This model aims to improve flows of information and response time by linking clinical care, vector and virus surveillance, and environmental surveillance, while engaging community members and stakeholders from sectors not typically involved in disease control (e.g., educators, waste management decision makers, business owners). This transition is ongoing in Ecuador, requiring significant intersectoral coordination, community engagement, and knowledge of the local social-ecological system (SES) drivers that influence dengue transmission [12]. Through previous studies in Ecuador, we have begun to characterize this system, demonstrating that dengue risk is associated with local socio-demographic factors and climate [13–15]. Community perceptions and responses, the focus of this study, are critical components of this system, since perceptions shape actions and thus govern behavioral responses and acceptance of shifts in policy and management.

The SES approach provides a useful research framework for understanding and identifying key local drivers of disease trransmission [16–20]. This problem-driven research approach is grounded in systems thinking, and focuses on the interactions among coupled human and natural systems that occur across a range of spatial, temporal and organizational scales (e.g., individual, community, society) [21]. Systems thinking allows the investigator to identify key policy leverage points in the system – places where decision makers and others can intervene to create desired change. Previous studies have demonstrated the effectiveness of similar approaches to study dengue and other public health issues [4, 5, 12, 22–26].

Here we present a novel application of the SES approach to investigate community perceptions, misconceptions, and local SES risk factors for dengue in Machala, Ecuador. We conducted this investigation in high risk communities located at the urban periphery and center, one year after the 2010 epidemic, and we compared findings between the two sites. This study contributes to an ongoing multi-year study to strengthen integrated dengue surveillance systems in Machala in partnership with the Ministry of Health and National Institute of Meteorology and Hydrology. The results of this study provide information that will inform the design and implementation of dengue control and surveillance interventions during this period of transition to a decentralized vector control program.

## Methods

### Study site and study population

Machala (population of 245,972) is the capital city of El Oro Province [7], and is a major port on the Pacific Coast, located 70 kilometers north of the Peruvian border. The economy in the region is based on primary production from agriculture (banana, cacao, coffee), fisheries, aquaculture (shrimp), and mining. Machala is typical of mid-sized cities in Latin America that experienced rapid, unplanned growth from 1960 to 1980, resulting in uneven access to piped water, garbage collection, and paved roads in the urban periphery. The socio-demographic characteristics of Machala have been described previously [13, 26].

**Figure 2:**
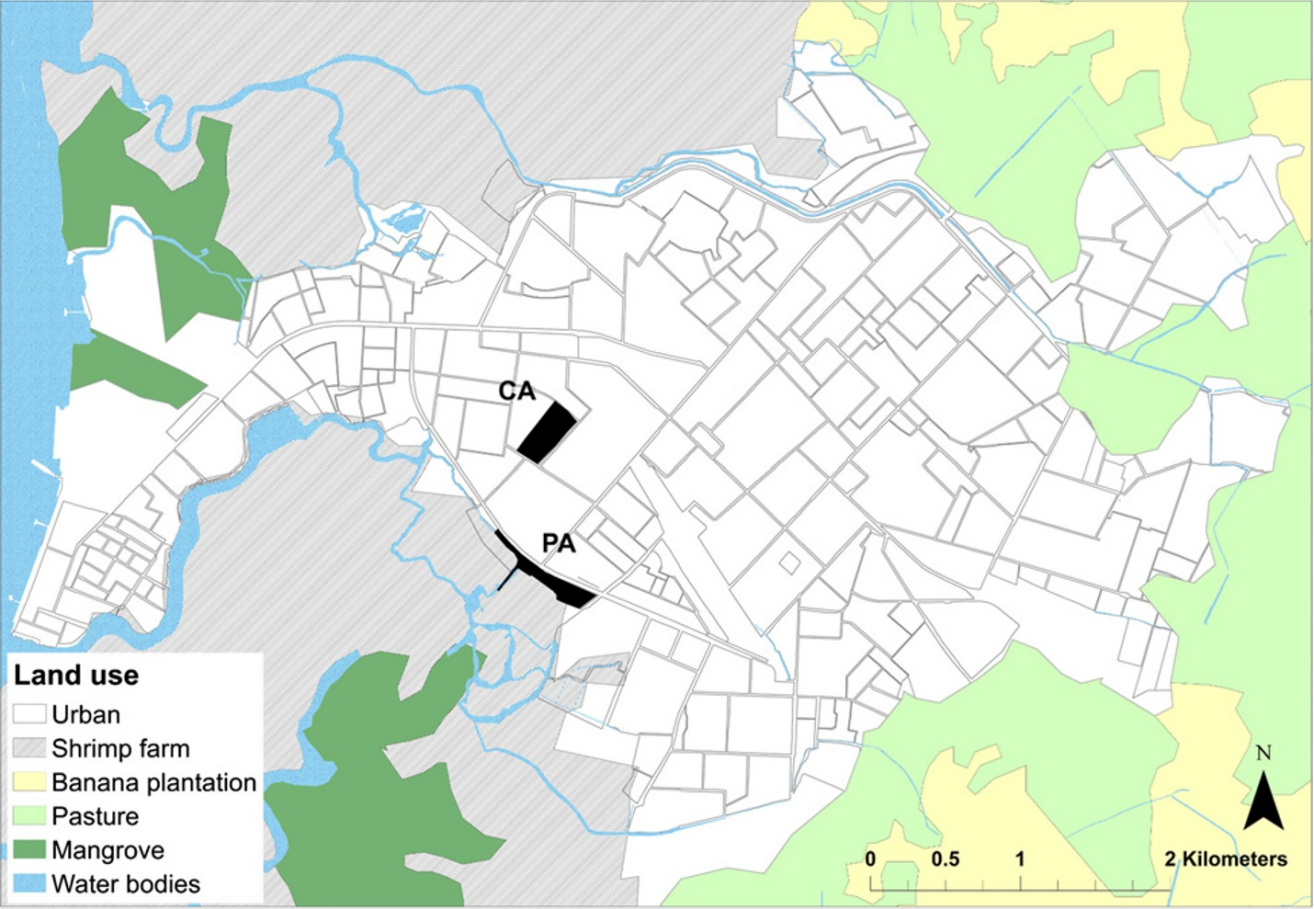
**Location of study sites in Machala, Ecuador.** Central area (CA) and peripheral area (PA) study sites indicated, with urban neighborhoods and land use. Land use map source: Ecuadorian Ministry of Agriculture, Livestock, Aquaculture and Fisheries (MAGAP), http://geoportal.magap.gob.ec/. Neighborhood map source: National Institute of Meteorology and Hydrology of Ecuador (INAMHI).

We conducted this investigation in two proximate (0.5 km apart) urban areas in Machala. The peripheral study area (PA) comprised two adjacent communities located at the southernmost edge of the city, Primero de Enero (population of 687) and Heroes de Jambeli (population of 388) (Figure 2). Some PA households were *invasiones* (e.g., properties without legal land tenure), limiting their access to municipal services. The central study area (CA) was comprised of the Veinte-cinco de Diciembre (population of 906) (Figure 2), an urban residential neighborhood, with recent improvements in infrastructure. Population estimates were derived from the 2010 national census in a previous study [15]. The study areas were among the most affected during the 2010 epidemic. Forty cases of dengue were reported from the PA (372 cases per 10,000 pop.) and sixteen cases were reported from the CA (177 cases per 10,000 pop.) according to Ministry of Health records [6].

### Research methods

This study was conducted from December 2010 to May 2011, in conjunction with a surveillance study that investigated the climatic and household factors influencing seasonal *Ae. aegypti* dynamics in these same communities [13].

#### Focus groups

We recruited community members to participate in focus group discussions in consultation with the presidents of each neighborhood council. People were selected on the basis of gender, community of residence, and a range of past experiences with dengue (some with dengue infections in their family, others without). An average of five to six people participated in each focus group (range 3 to 8 people), which were segmented by gender and neighborhood (2 neighborhoods from the PA, 1 CA neighborhood).

We facilitated semi-structured focus group discussions and causal diagram focus group discussions with groups of men and women from each neighborhood, for a total of 4 focus groups in the CA and 8 in the PA. The semi-structured focus group question guide included open-ended questions regarding people’s previous experiences with dengue, prevention practices, and the roles of the community and government in dengue control. Within two weeks the same groups reconvened to create causal (system) diagrams depicting the risk factors for dengue in their community. Causal diagrams allowed us to capture people’s perceptions of the connections among the social and environmental drivers influencing dengue transmission in their communities [25], allowing the investigator to define the “hypothesis space” and system boundaries [27]. Causal diagrams have been used by many researchers in the field of public health and development, with approaches ranging from wholly qualitative to complex mathematical models [25, 27–30]. We adopted an approach similar to the “problem tree analysis”, a participatory rural appraisal (PRA) tool to understand community perceptions of causality [31, 32].

All focus group discussions were held in the evening in a community meeting area and lasted between 60 and 90 minutes. Ministry of Health representatives were present at every meeting to address misconceptions and answer questions once the discussion was over. All discussions were tape-recorded and transcribed with permission from participants. Principal investigator AMSI facilitated the discussions, while co-investigators MS and TO acted as observers and notetakers.

We analyzed transcripts from semi-structured focus groups discussions and the text from causal diagrams to identify emergent themes using standard qualitative theme analysis methods based in grounded theory [33, 34]. AMSI manually coded transcripts from semi-structured focus groups (984 codes) and text from the causal diagrams (214 codes) in consultation with co-investigators VAL, SJR, and MJBC. To estimate the relative importance of each theme in each study area, we created a database of codes and themes in excel and tabulated the number of focus groups in which each theme emerged.

#### Household surveys

Following the completion of the focus groups, we designed household survey questions to further explore the themes that emerged, allowing investigators to triangulate quantitative and qualitative findings. Questions were also informed by our experiences with a prior pilot study on dengue led by co-investigator MJBC in Guayaquil, Ecuador, in 2008, and an ongoing multi-country study of dengue control interventions led by co-investigator EBA in Machala, Ecuador. Surveys were evaluated by vector control technicians and co-investigators at the Ministry of Health and field tested prior to use. We surveyed 79 households (39 PA households, 40 CA households) that were participating the ongoing vector surveillance study [13], where households were randomly selected to represent uniform distribution across each study area. The inclusion criterion was that a consenting adult at was home during the day throughout the surveillance study (November 2010 to June 2011). The head of the household or the responsible adult who resided in the home during the day was asked to respond to questions in a face-to-face interview that solicited information about dengue knowledge and perceptions, household demographics, vector control and water storage practices, and open-ended questions about the roles of the government and community. We compared survey responses from the two study areas using descriptive statistics in R (*i.e.*, Pearson’s Chi-squared test, Fisher’s Exact Test when values in the contingency table were less than 5, and Welch’s 2 sample two-sided independent t-test) [35].

#### Ethics statement

The investigation protocol was reviewed and approved by the Institutional Review Board of Syracuse University. Heads of households aged 18 years or older signed an informed consent form before participating in household surveys. Verbal and written consent was obtained from all participants in focus groups, who were all over the age of 18. No personal identifying information was collected.

## Results and discussion

**Figure 3:**
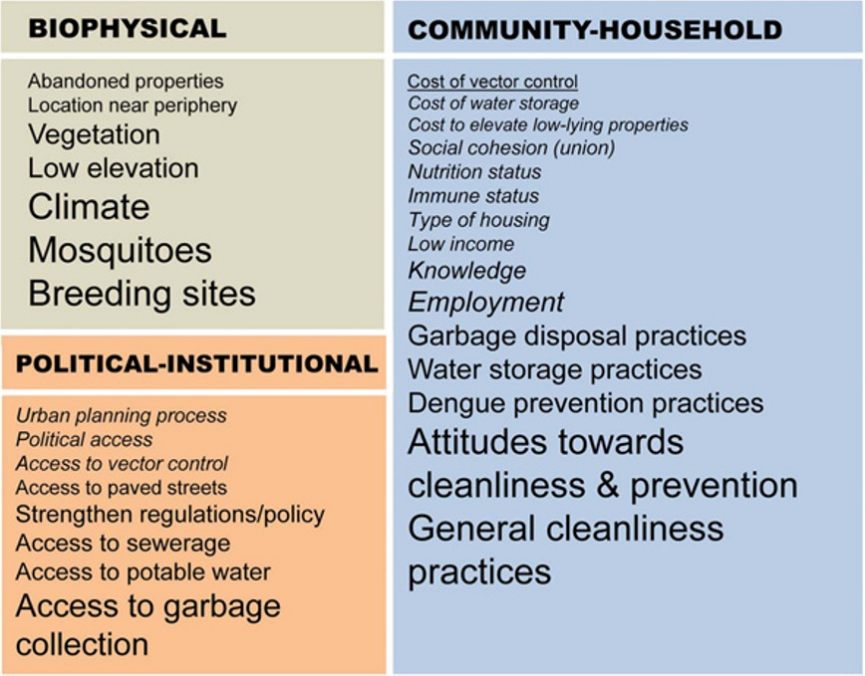
**Risk factors associated with dengue identified through a thematic analysis of causal diagrams.** Font size indicates the number of focus groups in which the theme emerged (range 1 to 6). Themes that emerged only from the peripheral area (PA) are in italics; themes only from the central area (CA) are underlined.

Household survey results are presented in Tables 1, 2 and 3. Findings from semi-structured focus group discussions are presented in the text, and themes are presented in Additional file 1: Table S1. Findings from the theme analysis of causal diagrams are presented in Figure 3 and Additional file 2: Table S2; emergent themes (n = 30) are shown in ascending order of importance (number of focus groups, range 1 to 6 out of 6).

**Table 1:** **Socio-demographic information of survey respondents (n = households)**

	Peripheral area (n = 39)		Central area (n = 40)		***p***value
Number of people in surveyed households	170		162		
People with self-reported previous dengue infection *(% of people in surveyed households)*	33	19%	32	20%	1
People with previous dengue infection who sought medical care *(% of infected people)* ^A^	25	76%	30	94%	0.082
Young family (mean age <35)	30	77%	20	50%	0.025
Female head of household	10	26%	12	30%	0.856
Head of household with post-secondary education	9	24%	12	32%	0.608
Head of household immigrated for work in the past 2 years	5	13%	8	20%	0.613
Head of household is currently employed or seeking work^A^	36	92%	34	85%	0.481
Access to sewerage^A^	30	77%	40	100%	0.002
Access to municipal garbage collection^A^	33	85%	40	100%	0.011
Piped water inside the house	20	51%	35	88%	0.001
Daily or weekly interruptions in the piped water supply^A^	15	39%	3	8%	0.001
Water storage in cisterns or covered elevated tanks^A^	25	66%	37	95%	0.001
Water stored in other containers	21	54%	9	23%	0.008

*p* values ≤0.05 indicate significant differences between the study areas; values were calculated using Pearson’s Chi-squared test with Yates continuity correction, unless otherwise indicated.

^A^ *p* values calculated by Fisher’s Exact Test.

**Table 2:** **Perceptions of dengue control from survey respondents (n = households)**

	Peripheral area (n = 39)		Central area (n = 40)		***p***value
*Challenges to vector control in the household*					
No challenges	17	44%	20	50%	0.73
Lack of information^A^	7	18%	0	0%	0.005
Economic factors	14	36%	10	25%	0.419
Lack of time^A^	5	13%	3	8%	0.481
Too many mosquitoes^A^	1	3%	2	5%	1
Other: lack of concern, health issues, type of housing^†^	5	13%	17	43%	
*Role of the government in dengue control*					
Chemical control	26	67%	21	53%	0.292
Public health education	11	28%	10	25%	0.946
Public utilities & services	19	49%	8	20%	0.014
Increase community interactions	7	18%	8	20%	1
Other: distribute mosquito nets, cut vegetation, chlorinate drinking water	4	10%	1	3%	
*Role of the community in dengue control*					
Become more united	18	46%	15	38%	0.793
Take preventative action in their own households	12	31%	15	38%	0.694
Ask for help from authorities	12	31%	7	18%	0.264
Organize community clean ups (*mingas*)^A^	4	10%	3	8%	0.713
Other: educate each other, can’t do anything, don’t know^†^	2	5%	5	12%	

*p* values ≤0.05 indicate significant differences between the study areas; values were calculated using Pearson’s Chi-squared test with Yates continuity correction, unless otherwise indicated.

^A^ *p* values calculated by Fisher’s Exact Test. ^†^Indicates multiple responses.

**Table 3:** **Mosquito control practices from survey respondents (n = households)**

	Peripheral area (n = 39)		Central area (n = 40)		***p***value
Clean garbage	39	100%	40	100%	1
Cut vegetation	35	90%	34	85%	1
Close windows & doors^A^	36	92%	28	70%	0.02
Cover containers with water	30	77%	34	85%	0.53
Eliminate standing water	31	79%	32	80%	1
Fumigation	30	77%	28	70%	0.658
Add chemicals to water to kill larvae	24	62%	26	65%	0.932
Use repellent	17	44%	23	58%	0.312
Screens on windows & doors	20	51%	19	48%	0.912
Pour burned diesel on floors & puddles	21	54%	12	30%	0.055
Burn incense or grass to make smoke	13	33%	14	35%	1
Other: mosquito nets, wash cistern, eat early, other insecticide^†^	8	21%	8	20%	1

*p* values ≤0.05 indicate significant differences between the study areas; values were calculated using Pearson’s Chi-squared test with Yates continuity correction, unless otherwise indicated.

^A^ *p* values calculated by Fisher’s Exact Test. ^†^Indicates multiple responses.

### Social-ecological risk factors

Through the analysis of causal diagrams, we found that the local risk factors for dengue included interrelated biophysical, political-institutional, and community-household factors (Figure 3). Climate was one of the most important biophysical risk factors (6 out of 6 focus groups), reflecting common knowledge that dengue risk was associated with temperature and rainfall, as shown in our previous studies [13, 14]. The most important political-institutional risk factors were associated with access to municipal public services and utilities (e.g., garbage collection, sewerage, piped water), indicating that people conceptualized dengue risk within the broader issues of urban development. Waste management was a major concern, especially in the urban periphery, where people lacked access to garbage collection services.

“The man who lives at the corner outside, instead of leaving the garbage out there, brings it in a cart and throws it in the canal. And he lives right at the curb. Imagine that! (PA participant)”.

The most important community-household risk factors were poor habits of cleanliness and attitudes regarding cleanliness and prevention (6 of 6 focus groups, Figure 3). People perceived that dengue risk in their community was associated with other people’s careless or apathetic attitudes, a concept that was captured, in part, by the phrase “*quemeimportismo*” (understood to mean an attitude of ‘*que me importa’* or ‘why should I care’).

“The neighbors don’t care and there is no way to say anything to them. They are not interested. For example, they don’t clean, don’t tidy up, and some say ‘if I get sick, if I am bitten, only then…’ I mean it’s their carelessness [*quemeimportismo*], sometimes they are not interested (CA participant)”.

“If I, in my house, take actions, but the next door neighbor doesn’t take action, then it’s all the same, because the mosquito will fly throughout the sector. So I think that *there* is the problem. There is still a lack of motivation. All of that is lacking (PA participant)”.

This finding supports findings by Mitchell-Foster (2013), who identified *quemeimportismo* as a central concept in community perceptions of dengue control in Machala, indicating “a pervasive sense of the futility of striving to improve conditions under the oppressive thumb of corruption and social issues” (pg. 98) [36].

Dengue presented a greater burden of disease in the PA compared to the CA during the 2010 outbreak, as indicated by the incidence rate ratio (IRR) of 2.1 (95% CI = 1.18, 3.76). Significantly more PA survey respondents (74%) were aware of cases of dengue in the community than CA respondents (50%) (*p* = 0.046, Chi-squared test), and most PA people (87%) reported that dengue was a problem in the community. Risk perception may have been elevated during this study due to the recent epidemic, which had overwhelmed the capacity of the public health system.

“There was a period of dengue when there were so many people, people piled on top of each other, and the hospital couldn’t take them (PA participant)”.

PA people identified a greater number of unique risk factors for dengue (Figure 3, Additional file 2: Table S2). People from both PA neighborhoods identified lack of political access, lack of access to vector control, low income, and lack of knowledge as risk factors. Factors unique to Heroes de Jambeli included the lack of access to the urban planning process, the cost to elevate low-lying properties, lack of social cohesion, and type of employment. Factors unique to the Primero de Enero included the cost of improved water storage, nutrition and immune status, type of housing. The cost of vector control was the only factor unique to the CA neighborhood, Veinte Cinco de Diciembre.

PA people perceived that they were neglected by government institutions (i.e., lack of political access in Figure 3), which increased their risk of dengue by limiting their access to public services and urban planning, and increasing their exposure to environmental contamination.

“This is an area where we have been marginalized and they [the authorities] have left us abandoned (PA participant)”.

“Here, I don’t see that we can prevent [dengue]… there is a strong contamination that is all around us, and this makes it difficult for me. (PA participant)”.

“We’re not going to wait for you [the authorities] to come do what we could do, but you also need to help us (PA participant)”.

Significantly fewer peripheral households had access to garbage collection, sewerage, and piped water inside the home, and a greater number of households reported interruptions in the piped water supply, necessitating water storage in containers around the home (*p* ≤0.05, Table 1). This disparity was greatest in areas that were *invasiones*, areas without legal land tenure. Accordingly, PA survey respondents identified a significantly greater number of government actions needed to reduce dengue in their communities (an average of 2.26 ± 0.15 (s.e.) actions in the PA versus 1.33 ± 0.16 actions in the CA, Student’s t-test, *p* <0.001), with significantly greater demand for access to public services, such as piped water and garbage collection (*p* = 0.014, Table 2).

In contrast, CA people felt empowered and well prepared to prevent dengue, thanks to the recent municipally supported infrastructure (*regeneración*) projects.

“The people have constructed sidewalks, they have organized communal clean-ups (*mingas*) to throw out all rubbish, and I think a lot has been done, not specifically to prevent dengue, no, but rather as a mechanism to elevate the quality of life (CA participant)”.

“They [the municipal government] have done a lot to protect us. Now it depends on us (CA participant)”.

Recent *regeneración* efforts in the CA were attributed, in part, to strong personal connections with local authorities, highlighting the importance of political access. Although the majority of CA (95%) survey respondents perceived that dengue was a problem in their community, focus group participants identified other important public health problems, such as alcoholism and drug abuse. Fewer CA respondents (60%) had received information about dengue and dengue prevention than PA respondents (82%) (*p* = 0.056, Chi-squared test). Despite recent improvements in infrastructure, a surveillance study conducted in 2010–2011 found that *Ae. aegypti* was more abundant in the CA than in the PA, especially during the rainy season [31]. This unexpected finding may be due to people who continued to store water as a backup water source despite improved access to piped water (i.e., a lag in behavioral change following rapid changes in urban infrastructure). These findings suggest that the *regeneración* efforts and lack of information may have lent people a false sense of security, causing them to underestimate their risk.

Our results indicate that the center-periphery social context plays an important role in community perceptions and responses to dengue risk, and should be considered in the design and implementation of interventions that target site-specific risk factors. These key differences include disparities in access to public services and economic resources, and perceptions of risk, empowerment, and political access. Our results also indicate that interventions may be more effective if they are framed within the broader development priorities that are specific to each community, such as legal land tenure and access to public services in the periphery versus the *regeneración* efforts in the central area. This approach would enhance the benefits that people receive and increase the likelihood that people will adopt new preventative behaviors [37]. By addressing dengue control as part of the broader issue of waste management, for example, the public sector on the Galapagos Islands of Ecuador implemented a successful campaign to reduce *Ae. aegypti.* The campaign focused on the elimination of tires, the potential use of tires as recyclable material, improvement in people’s quality of life, and benefits to local tourism. Similarly, vector control interventions in Asia that included waste management activities were able to more effectively reduce vector densities [12]. Interventions that target populations at the urban periphery could include the development of mosquito-proof public housing (similar to the project *Healthy Houses for Healthy Living* to prevent Chagas disease in Ecuador, M.J. Grijalva, *pers. comm.*), upgrading piped water infrastructure, subsidies or grants to improve water storage systems (e.g., for cisterns and pumps), and collaborations with large-scale, private shrimp and banana producers to improve waste management.

### Household dengue prevention

We found that mosquito control required a considerable investment of time and resources by households in both areas. Survey respondents said that they employed 7 to 8 mosquito control strategies, on average, including chemical control, elimination of larval habitat, and mosquito avoidance behaviors (Table 3). More PA respondents reported pouring diesel on the floor to repel mosquitoes (*p* = 0.054) and closing windows and doors (*p* = 0.02), mosquito avoidance practices that may reflect poorer housing quality. Economic factors were the most commonly mentioned barrier to dengue prevention, identified by 36% of PA respondents and 25% of CA respondents (Table 2). Focus group participants, especially from the PA, said that they struggled with the costs of insecticide, mosquito nets, and screens for windows, as indicated by the risk factors in Figure 3.

“As I see it, the difficulty is how we live. We live here in bamboo houses… No matter what we do, the mosquitoes enter and we get sick… What can we do in this case? (PA participant)”.

“I think that one of the difficulties, many times, is the economic situation of people… there are those of us who don’t have resources to buy a mosquito net… and we see many people who are extremely poor, who can buy their small bed or sleeping mat, but don’t have [anything] to protect themselves from the mosquitoes. This is a reason why there is dengue (PA participant)”.

Studies from dengue and malaria endemic regions have documented the high cost of household mosquito control [38, 39]. These findings highlight the importance of interventions that reduce the cost of household dengue prevention, such as free or low-cost insecticide impregnated screens or mosquito nets.

Women were largely responsible for dengue prevention and the overall health of their families, which presented additional challenges for working women.

*“*Sometimes we work, we get home late, sometimes tired, and sometimes we don’t have time – even though it doesn’t take much time (PA woman)”.

We found that 28% of households in this study were headed by women (Table 1). Our previous research in Machala showed that neighborhoods with a higher proportion of households headed by women were at greater risk for dengue infection [15]. These findings indicate the potential to develop dengue interventions that target working women and women heads of households. Parks and Lloyd [37] highlight the important role of women in social mobilization and behavioral impact interventions, which could include educational campaigns to encourage other members of the household to engage in prevention and family health care, greater engagement with women community leaders and women’s groups, and linking dengue interventions to income-generation activities for women.

We found that people’s perceptions of dengue and mosquito control reflected social stigmas associated with poverty, uncleanliness, and disease. Despite the economic barriers identified above, most people said in initial focus group discussions that dengue prevention was straightforward, and depended on each individual (generally women) to keep the house clean and free of standing water, regardless of income.

“Everything is clear. We each need to pay attention so that… our house is as clean as possible, cared for, to prevent dengue (PA participant)”.

“Cleanliness is something that does not have a cost, only [requires] the will of the person to do it (CA participant)”.

“We should be poor but clean (CA participant)”.

This rhetoric implied that people who had had dengue were unclean and careless, and since women were responsible for dengue control, this blame could be transferred to them. These findings suggest that public health messages that focus on clean homes for dengue prevention (e.g., *Patio Limpio* campaigns) may reinforce social stigmas and create misconceptions (see Knowledge Gaps) that reduce the effectiveness of the interventions, as shown in Mexico [40] and Puerto Rico [41]. Instead, public health messages should aim to reduce stigmas associated with dengue and poverty that could act as barriers to action, especially for communities in the urban periphery.

### Knowledge gaps

We identified three common dengue misperceptions that persist despite ongoing education campaigns by the Ministry of Health. PA people identified lack of knowledge as a risk factor and barrier to dengue prevention (Figure 3, Challenges to prevention in Table 2). Misconceptions act as barriers to dengue prevention in the household when they limit people’s ability to change their behavior. These findings indicate the need for public health messaging that targets local misconceptions and for ongoing evaluation of the effectiveness of the messaging.

1. People confused dengue with other mosquito- and water-associated diseases, such as cholera, typhoid, malaria, and general febrile illness.

“Once I got dengue, not specifically dengue, but actually malaria, which I think is somehow related (PA participant)”.

Other studies from Latin America and the Caribbean also found that people confused dengue with malaria and other febrile and respiratory diseases [41–45]. These findings suggest that self-reported dengue illness should be interpreted cautiously.

2) People were unsure how the mosquitoes became “contaminated” with dengue and whether the disease was contagious. There was no mention of a virus, bacteria or other microorganism. Some thought that the mosquitoes acquired dengue from contaminated waters, such as the sewerage canal, while others thought that the mosquitoes always carried the disease.

“They [the mosquitoes] deposit their eggs and from there comes the dengue (CA participant)”.

3) People were unaware that the dengue mosquito fed inside the home during the day, and they were confused as to whether the dengue mosquito emerged from “clean water” in containers around the home or “dirty water” in the environment. Some people claimed that they had been misinformed by the authorities, which added to their frustration and confusion,

“They told us that dengue, the mosquito, doesn’t breed in the pools of water, but rather inside the home… before they told us in the talks [at the clinic] that the mosquito reproduces in the puddles and tires with water and that they do not reproduce inside the house. So now we are disoriented, so how can we protect ourselves from dengue? (PA participant)”.

Studies from other countries identified similar misconceptions about *Ae. aegypti* [41, 44, 46]. They found that people tend to construct a general mental model of mosquito ecology that matches the ecology of the common culex mosquitoes, which are a nuisance, nocturnal, and breed in “dirty” water in the environment. These misconceptions reinforce our recommendation to avoid public health messages about clean versus dirty households for dengue prevention.

### Inter-institutional coordination and the community-government partnership

People in focus groups reported that the Ministry of Health and municipal government were the most important institutions engaged in dengue control; however, there had been limited inter-institutional coordination. Survey respondents reported that the primary roles of the Ministry of Health should be to provide chemical control and public health education, and the role of the municipal government was to provide public services and utilities (Table 2). It should be noted that the most important political-institutional risk factors (e.g., garbage collection, piped water, sewerage) were municipal services. Other studies that conducted a network analysis of dengue control in this region also found that municipal governments play a key role in dengue control [36]. These findings suggest that dengue control programs could be more effective through stronger collaborations with municipal governments, which are increasingly autonomous and powerful in countries shifting towards decentralized governance structures, as in Ecuador.

Only one third (32%) of survey respondents had participated in dengue control efforts. Focus group participants expressed frustration that they had not been able to effectively mobilize the community to prevent dengue, which they attributed to a lack of social cohesion (*desunión*) in the community.

“If we are not united we cannot achieve our objective, because they always say that unity is strength(PA participant)”.

When asked about the role of the community in dengue prevention, “become more united” was the most common response, reported by 46% of PA respondents and 38% of CA respondents (Table 2). The *desunión* was attributed to a lack of community leadership, individualistic attitudes and apathy, conflict between neighbors due to socioeconomic differences, and lack of support from government authorities.

“People are given to apathy. They are not interested [in attending community meetings]. They stay home and many times say, ‘No, I think this soap opera is more interesting’… It really is like that. (PA participant)”.

Other studies in Latin America also found that *desunión* was an important factor in community responses to dengue control [36, 42]. Social cohesion is especially important in the urban periphery, where self-organization and community initiative play an important role in securing legal land tenure by the municipality. Well-organized communities with strong leadership are likely to achieve legal tenure more quickly, and with that come the benefits of basic urban services and planned urbanization.

Focus group participants identified the need to foster a community-government partnership to improve the design and implementation of dengue prevention interventions.

“I think that the Ministry of Health and the Municipal government should… elaborate an intervention plan based on the environment and context of our neighborhood. This plan or project should be enriched by the support of community representatives… I think they can [all] benefit from the coordination, information, logistical support (CA participant)”.

Community organization and empowerment strategies could be employed to foster this partnership and engage communities in dengue control programs, as shown in other countries [12, 37, 47–49]. Community health workers could train community leaders to more effectively engage with local institutions, reducing barriers to political access and enabling them to articulate and lobby for their needs. This approach is especially critical for peripheral communities that are not united and have limited prior experience partnering with the public sector.

### Limitations

This study is limited by sampling in only high incidence communities at one time point, by the number of study participants, and by fewer focus groups in the CA than in the PA. Although these small groups of community members did not represent the whole community, their perceptions and experiences reflected the prevailing situation in central and peripheral urban areas of Machala. To better understand community perceptions and local risk factors, we would ideally sample a greater number of communities that represent a broader range of socio-demographic conditions and dengue incidence. This type of social-ecological community assessment should be an ongoing process to better understand how people’s perceptions and behaviors evolve as new interventions are implemented.

## Conclusions

There is a need for local, policy-relevant research that can be translated to strengthen the design, implementation, and evaluation of new dengue management strategies. The findings from this study contribute to a growing body of research in this area, highlighting the importance of social-ecological community assessments to identify priorities and goals of the community, misconceptions, resource limitations and other challenges to dengue prevention. Based on the findings of this study and our experience working in the field, we have identified the following key policy and management recommendations (leverage points) to inform the ongoing transition to a decentralized dengue control program in Ecuador and other dengue endemic countries:

- Social-ecological community assessments should be part of an ongoing adaptive management process, to better understand how people’s perceptions and behaviors evolve as new interventions are implemented.
- The center-periphery context (e.g., social cohesion, political access, resource limitations, access to services) should be considered in the design and implementation of interventions that target site-specific risk factors. Interventions may be more effective if they are framed within the broader development priorities that are specific to each community.
- Interventions that target marginalized populations at the urban periphery could include the development of mosquito-proof housing, upgrading piped water infrastructure, subsidies or grants to improve water storage systems (e.g., for cisterns and pumps), collaborations with the private sector to improve waste management, and free or low-cost insecticide impregnated screens or mosquito nets.
- Urban infrastructure improvements that could potentially reduce dengue risk (e.g., improved piped water) should be coupled with social mobilization and communication campaigns to create changes in behavior (e.g., water storage practices).
- Interventions that target working women and women heads of households could include educational campaigns to encourage other members of the household to engage in prevention and family health care, greater engagement with women community leaders and women’s groups, and linking dengue interventions to income-generation activities for women.
- Public health messages should target local misconceptions, avoid messages about clean versus dirty households, and aim to reduce the stigmas associated with dengue and poverty that could act as barriers to action. The effectiveness of the messaging should be regularly evaluated.
- Stronger inter-institutional collaborations with municipal governments could increase the effectiveness of dengue control programs, especially as programs become decentralized.
- Community organization and empowerment strategies could be employed to reduce barriers to political access in the urban periphery, and improve community engagement in dengue control.

## Electronic supplementary material

Additional file 1: Table S1: Themes from focus groups with semi-structured discussions. (DOCX 15 KB)

Additional file 2: Table S2: Risk factors associated with dengue identified through a thematic analysis of causal diagrams. Numbers indicate the number of focus groups in which the theme emerged. Two focus groups were conducted in each of the three communities, resulting in four focus groups in the peripheral area and two in the central area. Data were used to construct Figure 3. (DOCX 13 KB)
