# Supplementary material for: A social-ecological analysis of community perceptions of dengue fever and Aedes aegypti in Machala, Ecuador
Source: BMC Public Health. 2014 Nov 4;14:1135. doi: 10.1186/1471-2458-14-1135 (PMC4240812; doi:10.1186/1471-2458-14-1135)
Supplement: Supplementary file 1 — Additional file 1: Table S1: Themes from focus groups with semi-structured discussions. (DOCX 15 KB) [file 12889_2014_7246_MOESM1_ESM.docx]

| Table S1. Themes from focus groups with semi-structured discussions. |
| --- |
| Disease perceptions and actions |
| 1. Awareness of dengue in the community |
| 1. Dengue severity & symptoms |
| 1. Dengue transmission mechanism |
| 1. Mosquito ecology and nuisance |
| 1. Vulnerable groups |
| 1. Action taken when ill with dengue |
| Prevention perception and actions |
| 1. Responsbility/role of the household in dengue prevention |
| 1. Household actions taken to prevent dengue |
| 1. Challenges to dengue prevention in the household |
| Community and government responses |
| 1. Actions in the community to prevent dengue |
| 1. Barriers to action in the community |
| 1. Role of the community in dengue prevention |
| 1. Attitudes towards institutions |
| 1. Effectiveness of interventions by institutions |
| 1. Actions taken by institutions to prevent dengue in the community |
| 1. Role of institutions in dengue prevention in the community |
