# Supplementary material for: A social-ecological analysis of community perceptions of dengue fever and Aedes aegypti in Machala, Ecuador
Source: BMC Public Health. 2014 Nov 4;14:1135. doi: 10.1186/1471-2458-14-1135 (PMC4240812; doi:10.1186/1471-2458-14-1135)
Supplement: Supplementary file 2 — Additional file 2: Table S2: Risk factors associated with dengue identified through a thematic analysis of causal diagrams. Numbers indicate the number of focus groups in which the theme emerged. Two focus groups were conducted in each of the three communities, resulting in four focus groups in the peripheral area and two in the central area. Data were used to construct Figure 3. (DOCX 13 KB) [file 12889_2014_7246_MOESM2_ESM.docx]

| Table S2. Risk factors associated with dengue identified through a thematic analysis of causal diagrams. Numbers indicate the number of focus groups in which the theme emerged. Two focus groups were conducted in each of the three communities, resulting in four focus groups in the peripheral area and two in the central area. Data were used to construct Figure 3. | | | | |
| --- | --- | --- | --- | --- |
| **Risk factors** | **Peripheral Area** | | **Central Area** | **Total** |
| **Biophysical** | Heroes de Jambeli | Primero de Enero | 25 de Diciembre |  |
| Abandoned properties | 0 | 1 | 1 | 2 |
| Location near periphery | 1 | 0 | 1 | 2 |
| Vegetation | 0 | 2 | 2 | 4 |
| Low elevation | 1 | 1 | 1 | 3 |
| Climate | 2 | 2 | 2 | 6 |
| Mosquitoes | 2 | 2 | 2 | 6 |
| Breeding sites | 2 | 2 | 2 | 6 |
|  |  |  |  |  |
| **Political Institutional** |  |  |  |  |
| Urban planning process | 2 | 0 | 0 | 2 |
| Political access | 1 | 1 | 0 | 2 |
| Access to vector control | 1 | 1 | 0 | 2 |
| Access to paved streets | 0 | 1 | 1 | 2 |
| Strengthen regulations/policy | 2 | 0 | 1 | 3 |
| Access to sewerage | 0 | 1 | 2 | 3 |
| Access to potable water | 1 | 1 | 1 | 3 |
| Access to garbage collection | 2 | 1 | 2 | 5 |
|  |  |  |  |  |
| **Community-household** |  |  |  |  |
| Cost of vector control | 0 | 0 | 1 | 1 |
| Cost of water storage | 0 | 1 | 0 | 1 |
| Cost to elevate low-lying properties | 1 | 0 | 0 | 1 |
| Social cohesion | 2 | 0 | 0 | 2 |
| Nutrition status | 0 | 2 | 0 | 2 |
| Immune status | 0 | 2 | 0 | 2 |
| Type of housing | 0 | 2 | 0 | 2 |
| Low income | 1 | 1 | 0 | 2 |
| Knowledge | 1 | 2 | 0 | 3 |
| Employment | 1 | 0 | 0 | 1 |
| Garbage disposal practices | 2 | 0 | 2 | 4 |
| Water storage practices | 2 | 1 | 1 | 4 |
| Dengue prevention practices | 2 | 1 | 1 | 4 |
| Attitudes towards cleanliness and prevention | 2 | 2 | 2 | 6 |
| General cleanliness practices | 2 | 2 | 2 | 6 |
